# La detección tardía del deterioro neurológico agudo incrementa la letalidad por trauma craneoencefálico.

**DOI:** 10.7705/biomedica.4786

**Published:** 2020-03-30

**Authors:** Alexander Rodríguez, Eliana Cervera, Rafael Tuesca, Karen Flórez, Ricardo Romero, Pedro J. Villalba

**Affiliations:** 1 Departamento de Medicina, Universidad del Norte, Barranquilla, Colombia Universidad del Norte Departamento de Medicina Universidad del Norte Barranquilla Colombia; 2 Unidad de Cuidado Intensivo, Fundación Clínica Campbell, Barranquilla, Colombia Fundación Clínica Campbell Barranquilla Colombia

**Keywords:** traumatismos craneocerebrales, escala de coma de Glasgow, pronósticos, resultados de cuidados críticos, accidentes de tránsito, mortalidad, Craniocerebral trauma, Glasgow Coma Scale, prognosis, critical care outcome, accidents, traffic, mortality

## Abstract

**Introducción.:**

El trauma craneoencefálico es una de las principales causas de muerte y discapacidad en adultos jóvenes. Su gravedad se define según la escala de coma de Glasgow. Sin embargo, el deterioro neurológico agudo no siempre concuerda con la gravedad inicial indicada por la escala, lo que implica una subestimación de la magnitud real de la lesión.

**Objetivo.:**

Estudiar la correlación entre la gravedad inicial del trauma craneoencefálico según la escala de coma de Glasgow y la condición final del paciente, en el contexto de diferentes variables clínicas y de los hallazgos de la tomografía.

**Materiales y métodos.:**

Se analizó una cohorte retrospectiva de 490 pacientes con trauma craneoencefálico cerrado que requirieron atención en la unidad de cuidados intensivos de dos centros de tercer nivel de Barranquilla. La estimación del riesgo se estableció con la razón de momios *(odds ratio,* OR) y un intervalo de confianza (IC) del 95 %. Se utilizó un alfa de 0,05 como nivel de significación.

**Resultados.:**

El 41,0 % de los pacientes requirió intubación endotraqueal; el 51,2 % había presentado traumas inicialmente clasificados como moderados y, el 6,0 %, como leves. El retraso en la implementación de un tratamiento agresivo afectó principalmente a aquellos con trauma craneoencefálico moderado, en quienes la letalidad aumentó al 100 % cuando no se detectó a tiempo el deterioro neurológico y, por lo tanto, el tratamiento agresivo se demoró más de 4 a 8 horas. Por el contrario, la letalidad fue de menos de 20 % cuando se brindó el tratamiento agresivo en el curso de la primera hora después del trauma.

**Conclusiones.:**

El riesgo de letalidad del trauma craneoencefálico aumentó cuando el deterioro neurológico se detectó tardíamente y el tratamiento agresivo se inició después de transcurrida la primera hora a partir del trauma.

## Abstract (en)

**Introduction:**

Traumatic brain injury is a leading worldwide cause of death and disability in young people. Severity classification is based on the Glasgow Coma Scale. However, the neurological worsening in an acute setting does not always correspond to the initial severity suggesting an underestimation of the real magnitude of the injury.

**Objective:**

To study the correlation between the initial severity according to the Glasgow Coma Scale and the patient outcome in the context of different clinical and tomography variables.

**Materials and methods:**

We analyzed a retrospective cohort of 490 patients with closed traumatic brain injury requiring a stay in the intensive care unit of two third-level hospitals in Barranquilla. The risk was estimated by calculating the OR (95% CI). The significance level was established at an alpha value of 0.05.

**Results:**

Forty-one percent of all patients required orotracheal intubation; 51.2% were initially classified with moderate trauma and 6,0% as mild. The delay in the aggressive management of the traumas affected mainly those patients with traumas classified as moderate in whom lethality increased to 100% when there was delay in the detection of the neurological worsening and in the establishment of the aggressive treatment beyond 4 to 8 hours while the lethality in patients who received this treatment within the first hour reduced to <20%.

**Conclusions:**

The risk of lethality in traumatic brain injury increases with the delayed detection of neurological worsening in an acute setting, especially when aggressive management is performed after the first hour post-trauma.

El trauma craneoencefálico se define como la presencia de una disfunción encefálica causada por una fuerza externa que resulta en la pérdida o en la disminución del nivel de conciencia, amnesia anterógrada o retrógrada, déficit neurológico o cualquier alteración del estado mental en el momento del trauma ^1^. Como consecuencia de este, aparece una lesión primaria que es proporcional a las características mecánicas del impacto. Antes de que aparezcan las manifestaciones clínicas, se produce una cascada de procesos inflamatorios y lesiones secundarias que conllevan deterioro clínico y empeoran el pronóstico de los pacientes. Las herramientas actuales para determinar la gravedad de la lesión, entre ellas la escala de coma de Glasgow, no permiten evaluar los procesos moleculares que se desencadenan a partir del trauma.

En este estudio se analizaron las consecuencias de la demora del tratamiento agresivo oportuno debido a la poca sensibilidad de esta escala y el consecuente aumento de la morbimortalidad entre los pacientes con este tipo de trauma.

El trauma craneoencefálico es la principal causa de muerte y de discapacidad en personas menores de 40 años en los países en desarrollo ^2^. Según los *Centers for Disease Control and Prevention* (CDC) de los Estados Unidos, entre 1995 y 2001, 1,4 millones de personas sufrieron este tipo de trauma y, de ellas, el 3,6 % murió, el 17 % fue hospitalizado y el resto fue dado de alta ^3^. En el 2005, en los Estados Unidos, se registraron 3,17 millones de personas (1,1 % de la población total) que vivían con discapacidad a largo plazo como resultado de una lesión por trauma cerebral. Se desconoce el número de pacientes ambulatorios con trauma craneoencefálico o el de quienes no recibieron atención médica ^3^.

En el estudio prospectivo de la cohorte de nacimiento del norte de Finlandia, se encontró que el 3,8 % de la población había sido hospitalizado, por lo menos, una vez debido a un trauma craneoencefálico durante sus primeros 35 años de edad, en tanto que en la cohorte de nacimiento de Christchurch en Nueva Zelanda, el 31,6 % de la población había experimentado, por lo menos, un trauma de este tipo en los primeros 25 años de su vida y había requerido atención médica (hospitalización, cuidado en sala de emergencia o atención ambulatoria) ^4^.

Son pocas las publicaciones en las que se hace la caracterización epidemiológica del trauma craneoencefálico en Latinoamérica. En Brasil, Romeu, *et al.*^5^, informan que se han registrado, aproximadamente, 125.000 hospitalizaciones al año por este tipo de trauma, con una incidencia de 65,7 admisiones por cada 100.000 habitantes al año. La letalidad hospitalaria fue de 5,1 por 100.000 al año, y la tasa de mortalidad fue del 7,7 %, con un costo promedio anual de gastos hospitalarios de USD$ 70'960.000 y un costo promedio de USD$ 568 por paciente. En ese estudio, el grupo de 20 a 29 años de edad, con una de las frecuencias más altas de ingreso hospitalario por trauma creaneoencefálico, presentó el mayor número de muertes durante la hospitalización.

En el caso de Barranquilla, según el informe del Sistema de Vigilancia Epidemiológica para Causas Externas (SIVELCE) del 2012, 950 personas perdieron la vida en eventos relacionados con accidentes de tránsito entre el 2003 y el 2011, y el mayor número de muertes se registró en el 2006. Entre los peatones, se registró la prevalencia más alta, con 43 %, seguida por la de los motociclistas (tanto conductores como pasajeros), con 38 % ^6^.

Los accidentes de motocicleta son especialmente significativos. En el informe mencionado, la mayor tasa de accidentes del periodo se asoció con el uso de la motocicleta como método informal de transporte, lo que también ocurre en otros países de ingresos bajos y medios, como Brasil, Perú, Vietnam e India ^7^. Durante la última década, la adquisición y el uso de vehículos de motor de dos ruedas han tenido un incremento significativo en muchas ciudades de Latinoamérica. Según el Banco Mundial, en el 2002 había, aproximadamente, 7'810.500 ciclomotores y motocicletas, lo que equivale a cerca de 18 motocicletas por cada 1.000 habitantes. En Brasil, por ejemplo, la flota de motocicletas aumentó 14 veces entre 1990 y 2008. Además, hay evidencia de que las motocicletas usadas como transporte público en algunas ciudades medianas de Perú y Colombia han reemplazado todo el tráfico ^7^.

El incremento masivo del uso de vehículos de dos ruedas ha aumentado los accidentes causantes de trauma craneoencefálico, en los que el impacto biomecánico en las estructuras encefálicas causa daño en el tejido nervioso, fundamentalmente, las llamadas lesiones primarias y secundarias ^2^. Las primarias se definen como el conjunto de lesiones nerviosas y vasculares que aparecen inmediatamente como consecuencia de la agresión mecánica, determinadas por la transmisión de energía al propio tejido, y su consiguiente compresión y deformación. Estas afectan predominantemente a las neuronas, pero también, a las estructuras vasculares de las células gliales y cerebrales ^8^.

El mecanismo del trauma también se ha relacionado con los hallazgos en la tomografía, especialmente con los hematomas epidural y subdural, la hemorragia subaracnoidea y el hematoma intracraneal, que predominan en la lesión focal de contacto directo. En la lesión por aceleración o desaceleración difusa, son más frecuentes los fenómenos de daño axonal difuso y el edema cerebral ^2^. Independientemente de las diferencias en su curso y secuencia, todos los tipos de lesión cerebral traumática tienen en común un número limitado de reacciones bioquímicas destructivas ^9^, las cuales son progresivas o, por lo menos, pueden afectar el tejido cerebral progresivamente mediante procesos de retroalimentación viciosos. Por lo tanto, la lesión primaria tiende a producir un daño más grave que el directamente asociado con el impacto inicial. Esa secuencia de eventos que conduce al daño final se define como lesión secundaria, término que se refiere a eventos que abarcan desde el orden celular hasta el bioquímico ^10^.

De hecho, después del impacto inicial, las alteraciones causadas por la liberación de neurotransmisores, el aumento del calcio intracelular y la liberación de mediadores tóxicos, contribuyen al daño de la membrana celular ^11^. Todas estas reacciones a escala molecular determinan la aparición de alteraciones funcionales neuronales, como convulsiones, alteraciones pupilares y, especialmente, el síndrome de hipertensión intracraneal, el cual se produce por el aumento del volumen cerebral debido a la ocupación de lesiones extraaxiales o por el edema cerebral dentro de la bóveda craneal rígida ^11^. Estas manifestaciones pueden aparecer temprana o tardíamente después de la lesión inicial, lo que resulta en una disminución en la puntuación de la escala de coma de Glasgow en la fase aguda del trauma, disminución que, en este artículo, se ha denominado deterioro neurológico, que es la consecuencia clínica de todos los procesos moleculares secundarios a la lesión subyacente.

La presencia de deterioro neurológico no siempre está relacionada con la clasificación inicial de la gravedad determinada por la escala de coma de Glasgow *(Glasgow Coma Scale,* GCS), la cual clasifica el trauma craneoencefálico como leve (GCS: 14-15), moderado (GCS: 9-13) o grave (GCS: ≤ 8) ^12^. Aunque esta escala está diseñada para permitir una evaluación confiable de la gravedad del trauma, tiene limitaciones asociadas con su carácter cualitativo que, en muchos casos, llevan a subestimar la magnitud real del problema o, en otros, a su sobreestimación ^13^, lo que plantea la necesidad de hacer una clasificación temprana y objetiva de la gravedad del trauma que permita la implementación oportuna de tratamientos específicos y un seguimiento adecuado, para establecer el pronóstico con mayor sensibilidad y especificidad y ayudar, así, a disminuir la letalidad y las secuelas asociadas. Hay una gran cantidad de estudios destinados a desarrollar modelos predictivos tempranos en el trauma craneoencefálico ^14^, así como a incluir biomarcadores en los protocolos de manejo ^15^.

Previamente, nuestro grupo de investigación publicó una actualización enfocada en los potenciales biomarcadores del trauma craneoencefálico. Los biomarcadores determinados hasta la fecha consisten en moléculas generadas *de novo* por el proceso de inflamación y aquellas constitutivas que se liberan en las neuronas y las células gliales después del trauma. Estas moléculas se pueden cuantificar en la sangre total o en el líquido cefalorraquídeo ^16^.

En este contexto, el objetivo del presente trabajo fue la caracterización epidemiológica del trauma craneoencefálico en el área metropolitana de Barranquilla y la descripción de las posibles limitaciones del enfoque actual para la clasificación de su gravedad inicial, lo que podría conducir a la implementación tardía de los tratamientos específicos.

## Materiales y métodos

### Población de estudio

Se hizo un estudio retrospectivo de cohorte en dos centros hospitalarios de Barranquilla y su área metropolitana, en donde se recolectaron los datos demográficos y clínicos relacionados con el trauma craneoencefálico. Los centros seleccionados fueron el Hospital Universidad del Norte y la Fundación Clínica Campbell, instituciones de nivel de complejidad media y alta, respectivamente.

La información se obtuvo de las historias clínicas de los pacientes con diagnóstico de trauma craneoencefálico de cualquier gravedad en el momento de su ingreso, entre enero de 2012 y diciembre de 2014, y que requirieron atención en la unidad de cuidados intensivos en algún momento de la evolución posterior al trauma (n=490).

Se recopiló la información concerniente a edad, sexo, fecha y hora de ingreso, fecha y hora del trauma, fecha y hora de ingreso a la unidad de cuidados intensivos y de aplicación del tratamiento agresivo, fecha y hora del alta o el fallecimiento, puntaje en la escala de coma de Glasgow en el momento de la admisión y después de esta, estado general al ingreso, complicaciones asociadas, intervenciones, hallazgos en la tomografía computarizada (TC) de cráneo, datos de la mecánica del trauma y anteriores accidentes de tránsito sufridos por el paciente, así como el resultado de la lesión.

Los datos se depuraron inicialmente a partir de 2.392 historias clínicas de pacientes con diagnóstico de trauma de cualquier tipo, lo que resultó en la selección de 967 pacientes con trauma craneoencefálico de cualquier naturaleza: 579 tratados en la Fundación Clínica Campbell y 388 en el Hospital Universidad del Norte.

De este total, 490 cumplían con los criterios de inclusión: mayores de 13 años, diagnóstico de trauma craneoencefálico cerrado, ingreso a la unidad de cuidados intensivos en algún momento de su hospitalización y accidente ocurrido en el área metropolitana de Barranquilla. Se excluyeron los pacientes con trauma penetrante y los accidentes ocurridos por fuera del área geográfica mencionada, así como aquellos con lesiones en el cuero cabelludo por bajo impacto energético, es decir, causados por elementos contusos sin alteración de la conciencia.

La búsqueda se hizo con base en los diagnósticos relacionados con trauma de cráneo. El término de "lesiones en el cuero cabelludo por bajo impacto energético" fue acuñado por los autores para designar todos aquellos diagnósticos que no correspondían a un trauma craneoencefálico, sino a lesiones y heridas que no habían generado ninguna alteración del estado de consciencia, incluido el síndrome posterior a la conmoción. En el 0,4 % de los casos, no fue posible obtener el puntaje de la escala de Glasgow hecha en el momento del ingreso y, por lo tanto, se desconocía la gravedad.

### Análisis estadístico

Los datos se recopilaron utilizando un formulario publicado en la web y se organizaron en una base de datos Excel™ para su posterior análisis, utilizando el paquete estadístico SPSS 23.0™.

Los datos categóricos se expresaron en números absolutos y porcentajes. El análisis bivariado se hizo mediante la prueba de datos categóricos (prueba de ji al cuadrado) con un alfa de 0,05 como nivel de significación; en aquellos análisis en los que se encontraron valores menores de 5 en la prueba de ji al cuadrado, se utilizó la corrección de Yates para la prueba estadística y el valor de p. Las variables cuantitativas se resumieron en medianas y rangos intercuartílicos (RIC). La estimación del riesgo se estableció al calcular la razón de momios *(odds ratio,* OR) con un intervalo de confianza (IC) del 95 %.

### Consideraciones éticas

La metodología utilizada se basó en la recolección y el análisis de datos obtenidos a partir de las historias clínicas disponibles en las bases de datos de los hospitales de estudio. El proyecto fue aprobado por un comité de ética independiente antes de la obtención de las bases de datos, las cuales fueron codificadas con el fin de garantizar la confidencialidad y la protección de datos sensibles de los pacientes.

## Resultados

En el cuadro 1 se presenta la distribución según la gravedad de los casos de trauma craneoencefálico admitidos entre el 2012 y el 2014. Se evidenció una proporción más alta de hombres que de mujeres (6:1), lo cual probablemente está asociado con el mayor número de conductores hombres. La mediana de la edad fue de 35 años (RIC=28), y el trauma se presentó en mayor proporción en el grupo entre los 18 y los 29 años de edad (30,8 %), lo cual refleja el impacto de este tipo de lesiones en la población joven y económicamente productiva de la región. De hecho, en el cuadro 1 se constata que el 65,7 % de los casos tenía entre 18 y 49 años.

**Cuadro 1:** Distribución según la gravedad del trauma craneoencefálico en pacientes de dos hospitales de Barranquilla, entre 2012 y 2014

	Gravedad					
		Leve (% de fila)	Moderada (% de fila)	Grave (% de fila)	Sin datos (% de fila)	Total
Datos demográficos						
Grupo de edad (años) (n=490)	<18	3 (10,7)	18 (64,3)	7 (25,0)	0 (0,0)	28
	18-29	23 (15,2)	99 (65,6)	27 (17,9)	2 (1,3)	151
	30-39	18 (18,4)	59 (60,2)	20 (20,4)	1 (1,0)	98
	40-49	12 (16,4)	46 (63,0)	14 (19,2)	1 (1,4)	73
	50-64	11 (12,8)	62 (72,1)	11 (12,8)	2 (2,4)	86
	65 o más	16 (26,6)	29 (53,7)	9 (16,7)	0 (0,0)	54
Sexo	Masculino	70 (16,7)	265 (63,2)	79 (18,9)	5 (1,2)	419
(n=490)	Femenino	13 (18,3)	48 (67,6)	9 (12,7)	1 (1,4)	71
Etiología	Accidente de tránsito	74 (16,5)	292 (65,2)	77 (17,2)	5 (1,1)	448
(n=490)	Caídas	5 (20,8)	14 (58,3)	4 (16,7)	1 (4,2)	24
	Otros	4 (22,2)	7 (38,9)	7 (38,9)	0 (0,0)	18
Accidentes de tránsito	Carro	5 (25,0)	11 (55,0)	4 (20,0)	0 (0,0)	20
(n=448)	Motocicleta	26 (12,2)	150 (70,4)	35 (16,4)	2 (0,9)	213
	Otros	4 (21,1)	11 (57,9)	4 (21,1)	0 (0,0)	19
	Peatón	8 (10,5)	53 (69,7)	15 (19,7)	0 (0,0)	76
	Camión	0 (0,0)	2 (100,0)	0 (0,0)	0 (0,0)	2
	Sin datos					118
Rol del paciente en el vehículo (no se incluyen peatones)	Conductor	27 (15,2)	119 (66,9)	31 (17,4)	1 (0,6)	178
	Pasajero de motocicleta	3 (6,3)	39 (81,3)	5 (10,4)	1 (2,1)	48
	Pasajero	4 (20,0)	11 (55,0)	5 (25,0)	0 (0,0)	20
(n=254)	Sin datos					8
Embriaguez	Sí	8 (12,7)	44 (69,8)	10 (15,9)	1 (1,6)	63
(n=490)	No	46 (14,6)	212 (67,3)	55 (17,5)	2 (0,6)	315
	No data					112
Condición final (n=490)						
Dado de alta		72 (19,6)	256 (69,8)	36 (9,8)	3 (0,8)	367
Muerte		2 (2,7)	31 (41,9)	39 (52,7)	2 (2,7)	74
Referido		8 (19,5)	20 (48,8)	12 (29,3)	1 (2,4)	41
Sin datos						8
Hallazgos en la primera tomografía computarizada						
Hematoma subdural		0 (0,0)	18 (90,0)	2 (10,0)	0 (0,0)	20
Hemorragia subaracnoidea		12 (11,8)	70 (68,6)	20 (19,6)	0 (0,0)	102
Hematoma epidural		5 (21,7)	17 (73,9)	1 (4,3)	0 (0,0)	23
Dos hallazgos		3 (6,8)	24 (54,5)	17 (38,6)	0 (0,0)	44
Tres hallazgos		0 (0,0)	3 (37;5)	4 (50,0)	1 (12,5)	7
Tratamiento						
Intubación endotraqueal		12 (6,0)	103 (51,2)	82 (40,8)	4 (2,0)	201
Relajantes neuromusculares		4 (7,1)	27 (48,2)	23 (41,1)	2 (3,6)	56
Manitol		42 (15,2)	183 (66,1)	51 (18,4)	1 (0,4)	277
Solución salina hipertónica		13 (8,5)	94 (61,4)	43 (28,1)	3 (2,0)	153
Craniotomía		14 (15,1)	59 (63,4)	18 (19,4)	2 (2,1)	93
Craniectomía descompresiva		0 (0,0)	4 (80,0)	1 (20,0)	0 (0,0)	5

El hallazgo más frecuente en la TC de ingreso fue la hemorragia subaracnoidea (52,0 %), seguido del hematoma epidural (11,7 %) y del hematoma subdural (10,2 %). Se presenta, asimismo, el número de pacientes que recibieron medidas generales o tratamiento agresivo para evitar la hipertensión intracraneal, según lo indicado por la clasificación inicial de la gravedad del trauma. Los tratamientos agresivos indicados para este tipo de trauma incluyen intubación endotraqueal, sedación y analgesia, bloqueo neuromuscular, craneotomía y craniectomía. Otras medidas generales incluyen la tratamiento hiperosmolar con manitol o solución salina hipertónica, y el soporte vasoactivo ^12^.

El 41,0 % de los pacientes (cuadro 1) requirieron intubación endotraqueal, de los cuales el 51,2 % había sido clasificado inicialmente con trauma moderado, y el 6,0 % con trauma leve, en tanto que el 40,8 % presentaba trauma grave. En cuanto a la condición final, el 74,9 % fue dado de alta y el 15,1 % falleció.

Al analizar el número de muertes por subgrupos según la gravedad, el 2,7 % correspondió a traumas leves, el 41,9 % a moderados y el 52,7 % a graves, es decir, hubo un número importante de pacientes cuya clasificación inicial y manejo subsecuente habrían sido inadecuados. En el 8,4 % de los casos no se registró la información sobre la condición final de los pacientes porque fueron referidos a otras instituciones durante su hospitalización.

Cabe señalar que el 32,7 % de los casos inicialmente clasificados con trauma moderado requirieron intubación endotraqueal, lo que indica que en algún punto de su evolución presentaron deterioro neurológico, entendido este como una caída en el puntaje de la escala de Glasgow de ≤8 en las primeras 72 horas a partir del momento del trauma. Todos los pacientes en este grupo recibieron tratamiento hiperosmolar, pero hubo un menor uso de solución salina hipertónica (29,8 %) en comparación con el grupo con trauma grave (100 %), en tanto que el uso de manitol (58,4 %) y las cirugías craneales (20,0 %) se presentaron en una proporción similar en ambos grupos.

**Figura 1:**
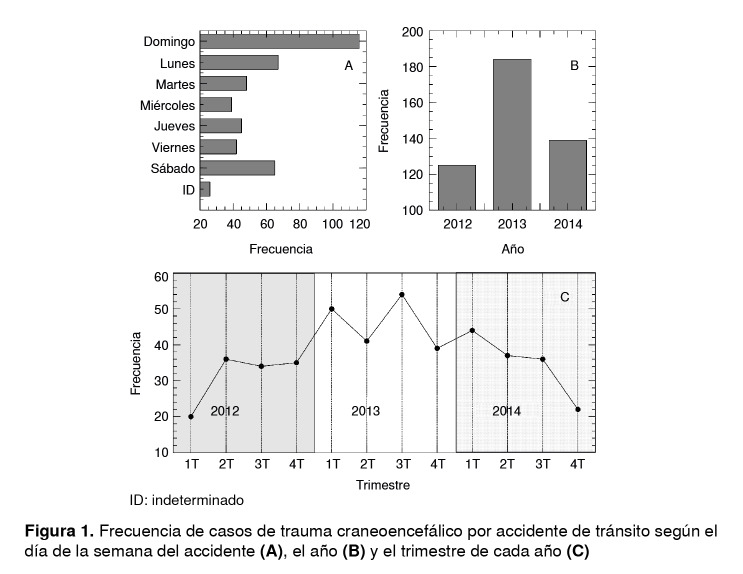
Frecuencia de casos de trauma craneoencefálico por accidente de tránsito según el día de la semana del accidente **(A),** el año **(B)** y el trimestre de cada año **(C)**

En el cuadro 1, también se puede apreciar cómo la mayoría de los casos de trauma fueron consecuencia de accidentes de tránsito (91,4%) y que los motociclistas fueron el grupo más afectado (47,5 %). En el 12,8 % de los casos se informó que los pacientes se encontraban en estado de embriaguez en el momento del trauma; sin embargo, es importante enfatizar que estos datos son completamente subjetivos, ya que los protocolos locales de manejo del trauma craneoencefálico no incluyen la alcoholemia como prueba de rutina y en muchos casos esta variable no se informa. En la figura 1A se aprecia que la frecuencia de este tipo de trauma aumentó a medida que aumentó la de los accidentes de tránsito, especialmente los fines de semana (sábado y domingo) y los lunes, días en que se registra mayor consumo de alcohol entre la población general.

Además, el 14,3 % del grupo con trauma leve también requirió intubación endotraqueal, el 16,7 % se sometió a craneotomía y más de la mitad recibió algún tipo de tratamiento hiperosmolar, con predominio del manitol (50,0 %). Estos datos reflejan que una proporción significativa de los pacientes desarrolló deterioro neurológico a pesar de que el puntaje en la escala de Glasgow en el momento del ingreso correspondió a un nivel de gravedad en el rango de leve a moderado. Se observó una fuerte asociación entre la presencia de deterioro neurológico en casos de trauma moderado y la muerte (p<0,001), con una proporción de muertes de 35,3 % en este grupo (cuadro 2).

**Cuadro 2:** Asociación independiente entre los hallazgos más importantes en la tomografia, las principales complicaciones y la muerte

	Muerte (%)	OR (IC _95%_)	χ^2^	p
Hallazgos en la TC				
Solo hematoma subdural	25,0	15,0 (2,66-84,48)	10,97	0,001
Solo hematoma epidural	4,3	2,04 (0,17-23,59)	0,02	0,49
Solo hemorragia subaracnoidea	12,7	6,72 (1,47-30,67)	6,36	0,006
Hematoma subdural y hemorragia subaracnoidea	10,0	5,19 (0,89-32,70)	1,93	0,08
Hematoma epidural y hemorragia subaracnoidea	10,0	5,0 (0,41-60,68)	0,16	0,26
hematoma subdural y hematoma epidural	0,0	NS	NS	NS
Hematoma subdural, hematoma epidural y hemorragia subaracnoidea	0,0	NS	NS	NS
Complicaciones principales				
Sepsis (cualquier foco)	28,6	4,75 (2,21-10,19)	18,68	0,0002
Choque séptico (cualquier foco)	41,9	25,63 (5,30-123,9)	24,00	0,000
Neumonía	42,9	14,26 (4,11-49,33)	23,11	0,0001
Deterioro neurológico	35,3	5,41 (3,09-9,48)	40,34	<0,001
Otras complicaciones				
Choque neurogénico	100,0	55,40 (15,10-203,21)	86,44	<0,001
Necesidad de traqueotomía	18,1	7,12 (1,63-31,21)	5,59	0,02
Intervenciones				
Relajación neuromuscular	25,6	4,02 (2,11-7,67)	18,26	0,000
Craniectomía descompresiva	4,2	7,71 (1,26-46,98)	3,99	0,04

NS: no significativo

En la figura 1B se aprecia un aumento sustancial de la frecuencia del trauma craneoencefálico secundario a accidentes de tránsito en el período comprendido entre el 2012 y el 2013, seguido de una disminución significativa en el 2014, lo que contrasta con el constante incremento en la venta de motocicletas durante ese mismo período ^17^. La disminución observada en la proporción de estos casos comenzó en el cuarto trimestre de 2013 (figura 1C) y podría asociarse con la implementación de la ley de la alcoholemia en Colombia (Ley 1696 del 19 de diciembre de 2013) ^18^, la cual establece sanciones penales y administrativas por conducir bajo la influencia del alcohol u otras sustancias psicoactivas.

Las complicaciones más frecuentes durante la hospitalización fueron la sepsis de cualquier origen (9,0 %), el choque séptico (3,3 %) y el choque neurogénico (3,7 %). En el cuadro 2, se presenta la asociación entre estas complicaciones, los hallazgos en la TC inicial y la muerte del paciente. La presencia de hematoma subdural o hemorragia subaracnoidea en la TC de admisión, se asoció significativamente con la muerte (p<0,001 y p<0,006, respectivamente), lo cual no ocurrió en los casos de hematoma epidural (p=0,49). Esta observación concuerda con estudios previos ^19^ y podría correlacionarse con el hecho de que los hematomas epidurales tienen mejores pronóstico y recuperación después del drenaje quirúrgico al ser una lesión que ocupa espacio, localizada por fuera de las meninges; además, el sangrado es de origen arterial, lo cual constituye un signo de alarma crítica para la rápida intervención de los neurocirujanos.

**Figura 2:**
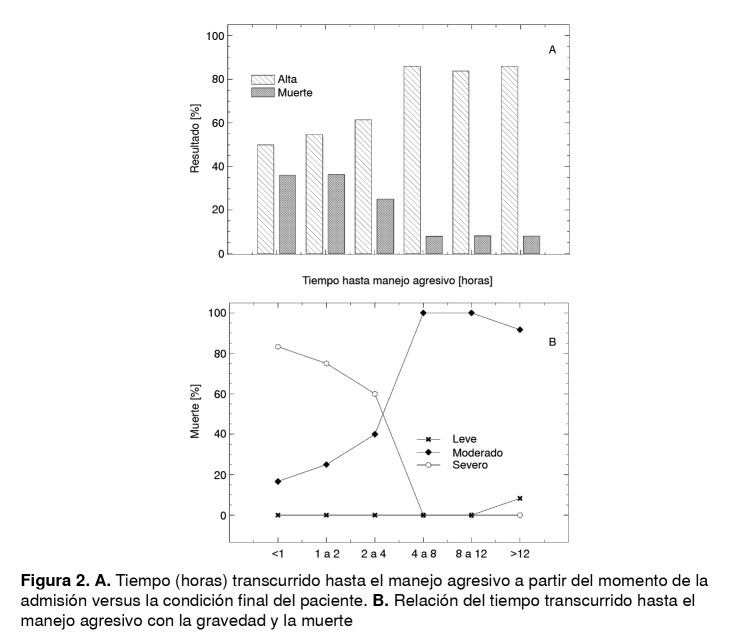
A. Tiempo (horas) transcurrido hasta el manejo agresivo a partir del momento de la admisión versus la condición final del paciente. B. Relación del tiempo transcurrido hasta el manejo agresivo con la gravedad y la muerte

Cuando se calculó el tiempo transcurrido entre el ingreso y el inicio del tratamiento agresivo, se observó que, en el grupo en el que dicho tiempo fue de menos de una hora, murieron 18 de 50 pacientes, es decir, el 36,0 %, en tanto que la letalidad en los otros grupos fue igual o inferior (figura 2B), lo cual sería contradictorio, pues se esperaría que, a medida que dicho tiempo disminuye, la tasa de supervivencia aumente. Sin embargo, la mayor letalidad observada en el grupo tratado agresivamente en las primeras dos horas, se debe a la gravedad del trauma, pues el tratamiento agresivo se recomienda para puntuaciones de menos de 8 en la escala de Glasgow, es decir, para el trauma craneoencefálico grave, en el cual se espera una mayor letalidad.

**Figura 3:**
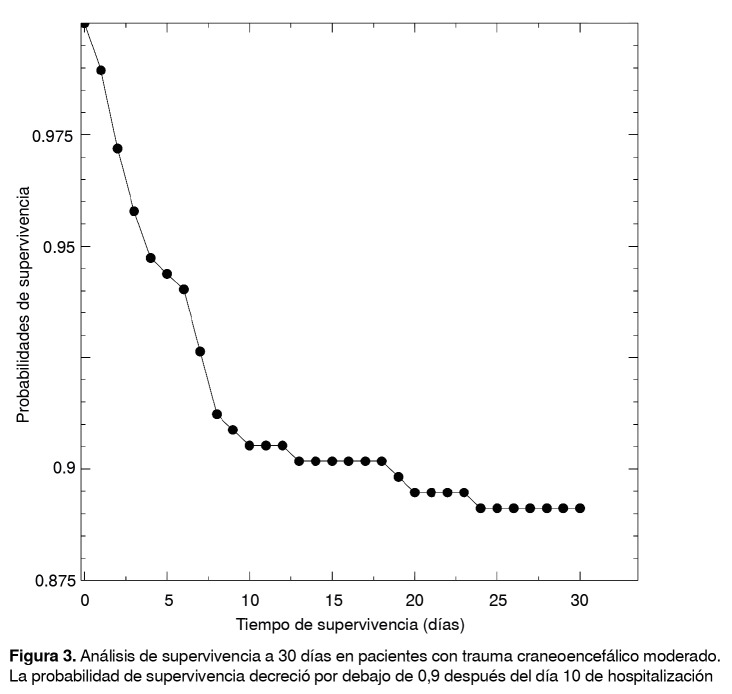
Análisis de supervivencia a 30 días en pacientes con trauma craneoencefálico moderado. La probabilidad de supervivencia decreció por debajo de 0,9 después del día 10 de hospitalización

Esta observación se hace evidente cuando se analiza cada subgrupo de gravedad por separado en función del tiempo transcurrido entre el ingreso y el tratamiento agresivo, y la condición final del paciente, como se observa en la figura 2A. Se demuestra así que el acentuado efecto de la demora en la implementación de medidas agresivas afecta principalmente al grupo de pacientes con trauma inicialmente clasificado como moderado, grupo en el que quienes fueron tratados en el curso de la primera hora presentaron una letalidad de menos de 20,0 %, con un notable aumento de la letalidad a medida que aumentaba el tiempo de instauración del tratamiento agresivo, y una letalidad cercana al 100 % cuando dicho tiempo excedió las 4 a 8 horas. Además, el análisis de Kaplan-Meier evidenció que la probabilidad de supervivencia disminuyó por debajo de 0,9 cuando la hospitalización se prolongaba más de 10 días (figura 3).

En cuanto a las principales complicaciones, se observó un aumento significativo de la letalidad cuando se presentó choque séptico (OR=47,48; IC_95%_ 5,14-4,37) y choque neurogénico (OR=55,40; IC_95%_ 15,10-203,21). Más específicamente, en el grupo con trauma moderado, el 18,2 % requirió medicamentos vasoactivos para el choque de cualquier origen y el 83,9 % falleció durante el tratamiento (cuadro 3).

**Cuadro 3:** Proporción de intervenciones específicas para el manejo de la hipertensión endocraneana en pacientes con trauma craneoencefálico moderado que desarrollaron deterioro neurológico y proporción de muertes por subgrupo

Intervención	Trauma craneoencefálico moderado y deterioro neurológico (%)	Muertes (%)
Intubación endotraqueal	100,0	32,9
Soporte vasoactivo	83,9	19,2
Manitol	58,1	58,5
Solución salina hipertónica	71,0	30,0
Craniotomía	29,0	18,8
Craniectomía descompresiva	6,5	1,3
Bloqueadores neuromusculares	19,4	8,6

En cuanto al tratamiento hiperosmolar, se observó una fuerte asociación entre el uso de solución salina hipertónica y la muerte (p<0,001), lo que no sucedió con el uso de manitol (p<0,94). Este hallazgo se explicaría por el hecho de que, en los protocolos de manejo de los hospitales del estudio, se prefiere el uso de solución salina hipertónica para el trauma craneoencefálico grave, en tanto que el manitol se prefiere para los traumas leves a moderados. Así, el 58,5 % de los pacientes con traumas moderados recibió manitol y, el 30,0 %, solución salina hipertónica, y de estos, el 58,1 % y el 71,0 %, respectivamente, murió en cada subgrupo (cuadro 3). La decisión ulterior de usar solución salina hipertónica en los casos de trauma moderado se basa con frecuencia en el deterioro neurológico, como se establece en los protocolos de los hospitales de estudio, lo que también explicaría el mayor número de muertes en el subgrupo tratado con solución salina hipertónica.

La necesidad de bloqueadores neuromusculares durante la administración de respiración mecánica asistida, también se correlacionó con un aumento de la letalidad (OR=4,03; IC_95_% 3,26-4,98), ya que este es sugestivo de hemorragia endocraneal y trauma craneoencefálico grave. Además, la decisión de practicar una craniectomía también se asoció con la muerte (OR=7,71; IC_95_% 1,46-40,81), lo que puede atribuirse al hecho de que, por lo general, esta es una intervención tardía en pacientes con hemorragia endocraneal que no mejoran con el tratamiento médico o temprano cuando se basa en los hallazgos específicos en la TC de ingreso indicativos de hemorragia endocraneal grave, que ya, *per se,* indican un mal pronóstico. Por el contrario, la asociación con la muerte no fue significativa en el caso de craneotomías para el drenaje de lesiones que ocupan espacio (p<0,94), lo que podría explicarse por el hecho de que, en muchos casos, esta técnica se emplea tempranamente a partir de hallazgos de lesiones que ocupan espacio sin evidencia de hemorragia endocraneal grave en la tomografía.

## Discusión

El hallazgo de que una proporción significativa de pacientes inicialmente clasificados con trauma craneoencefálico leve o moderado requirió intervenciones específicas para trauma grave por deterioro neurológico, sugiere que la escala de coma de Glasgow no es una herramienta lo suficientemente sensible para la clasificación inicial de la gravedad, como se ha descrito previamente en otros estudios ^13^. Esta observación debe correlacionarse con la presencia de varias manifestaciones fisiopatológicas después del trauma cerebral, las cuales constituyen los verdaderos factores determinantes de la gravedad del trauma, aunque tales procesos no siempre son clínicamente visibles ^20^. Por esta razón, su evolución conduce eventualmente a hipertensión endocraneal, pero el daño real queda oculto clínicamente, lo cual contribuye al retraso del tratamiento agresivo que, actualmente, solo está indicado cuando el puntaje es de 8 o menos en la escala de Glasgow ^12^.

Los pacientes con deterioro neurológico tuvieron un riesgo significativamente mayor de muerte en comparación con aquellos que no lo presentaron (OR=5,41; IC_95_% 3,09-9,48). Cuando el tratamiento agresivo se inició después de la primera hora, el riesgo de muerte casi se duplicó, lo que sugiere que la intervención agresiva se retrasó probablemente por la subestimación de la gravedad real. Asimismo, la presencia de hemorragia subaracnoidea o hematoma subdural en la primera TC se asoció significativamente con un mayor riesgo de muerte (OR=6,72 e IC_95_% 1,4730,67 y OR=15,0 e IC_95_% 2,66-84,48, respectivamente). (OR=2,04; IC_95_% 0,1723,59), probablemente debido a la naturaleza anatómica de este hematoma, ya que es externo al encéfalo (fuera de la duramadre) y genera una menor inflamación del cerebro, además de que representa una urgencia quirúrgica, lo que podría ayudar a reducir el tiempo transcurrido hasta la decisión de intervenir quirúrgicamente ^22^.

El objetivo del estudio fue señalar la necesidad de brindar un tratamiento según las circunstancias de cada paciente, más allá de la clasificación inicial del trauma basada en la escala de Glasgow debido a las limitaciones que dicha escala presenta. El someter a los pacientes a intervenciones agresivas implica la posibilidad de que se presenten efectos adversos y complicaciones, por lo cual no debe optarse por la intubación endotraqueal y la asistencia respiratoria mecánica invasiva de manera generalizada en todo caso de trauma moderado. Las guías de manejo aceptadas internacionalmente solo proponen un manejo agresivo en la población con trauma craneoencefálico grave ^12^, pero no hacen referencia a las intervenciones en los pacientes con trauma moderado y en riesgo de sufrir deterioro neurológico.

El análisis de estos datos permite concluir que el riesgo de letalidad en pacientes con trauma craneoencefálico aumenta con ciertos factores, como la presencia de hemorragia subaracnoidea o de hematoma subdural en la TC inicial, la necesidad de intubación endotraqueal y, especialmente, la implementación de un tratamiento agresivo después de la primera hora en casos con deterioro neurológico. También, habría limitaciones de la escala de coma de Glasgow en el diagnóstico precoz de la gravedad, lo que respalda el desarrollo de una estrategia cuantitativa, más objetiva y sensible, para la clasificación inicial de la gravedad de este tipo de trauma ^22^.

Nuestro grupo de investigación trabaja actualmente en el desarrollo de métodos para cuantificar biomarcadores en plasma ^15^, empleando una técnica rápida de medición que pueda utilizarse tempranamente en la sala de urgencias o, incluso, durante la evolución misma del trauma ^16^.
